# Adhesive and Migratory Effects of Phosphophoryn Are Modulated by Flanking Peptides of the Integrin Binding Motif

**DOI:** 10.1371/journal.pone.0112490

**Published:** 2014-11-14

**Authors:** Shigeki Suzuki, Seiji Kobuke, Naoto Haruyama, Hiroaki Hoshino, Ashok B. Kulkarni, Fusanori Nishimura

**Affiliations:** 1 Department of Dental Science for Health Promotion, Hiroshima University Institute of Biomedical and Health Sciences, Hiroshima, Japan; 2 Section of Orthodontics and Dentofacial Orthopedics, Faculty of Dental Science, Kyushu University, Fukuoka, Japan; 3 Functional Genomics Section, Laboratory of Cell and Developmental Biology, National Institute of Dental and Craniofacial Research, National Institutes of Health, Bethesda, Maryland, United States of America; 4 Section of Periodontology, Division of Oral Rehabilitation, Faculty of Dental Science, Kyushu University, Japan; Thomas Jefferson University, United States of America

## Abstract

Phosphophoryn (PP) is generated from the proteolytic cleavage of dentin sialophosphoprotein (DSPP). Gene duplications in the ancestor dentin matrix protein-1 (DMP-1) genomic sequence created the DSPP gene in toothed animals. PP and DMP-1 are phosphorylated extracellular matrix proteins that belong to the family of small integrin-binding ligand N-linked glycoproteins (SIBLINGs). Many SIBLING members have been shown to evoke various cell responses through the integrin-binding Arg-Gly-Asp (RGD) domain; however, the RGD-dependent function of PP is not yet fully understood. We demonstrated that recombinant PP did not exhibit any obvious cell adhesion ability, whereas the simultaneously purified recombinant DMP-1 did. A cell adhesion inhibitory analysis was performed by pre-incubating human osteosarcoma MG63 cells with various PP peptides before seeding onto vitronectin. The results obtained revealed that the incorporation of more than one amino acid on both sides of the PP-RGD domain was unable to inhibit the adhesion of MG63 cells onto vitronectin. Furthermore, the inhibitory activity of a peptide containing the PP-RGD domain with an open carboxyl-terminal side (H-^463^SDESDTNSESANESGSRGDA^482^-OH) was more potent than that of a peptide containing the RGD domain with an open amino-terminal side (H-^478^SRGDASYTSDESSDDDNDSDSH^499^-OH). This phenomenon was supported by the potent cell adhesion and migration abilities of the recombinant truncated PP, which terminated with Ala^482^. Furthermore, various point mutations in Ala^482^ and/or Ser^483^ converted recombinant PP into cell-adhesive proteins. Therefore, we concluded that the Ala^482^-Ser^483^ flanking sequence, which was detected in primates and mice, was the key peptide bond that allowed the PP-RGD domain to be sequestered. The differential abilities of PP and DMP-1 to act on integrin imply that DSPP was duplicated from DMP-1 to serve as a crucial extracellular protein for tooth development rather than as an integrin-mediated signaling molecule.

## Introduction

**Figure 1:**
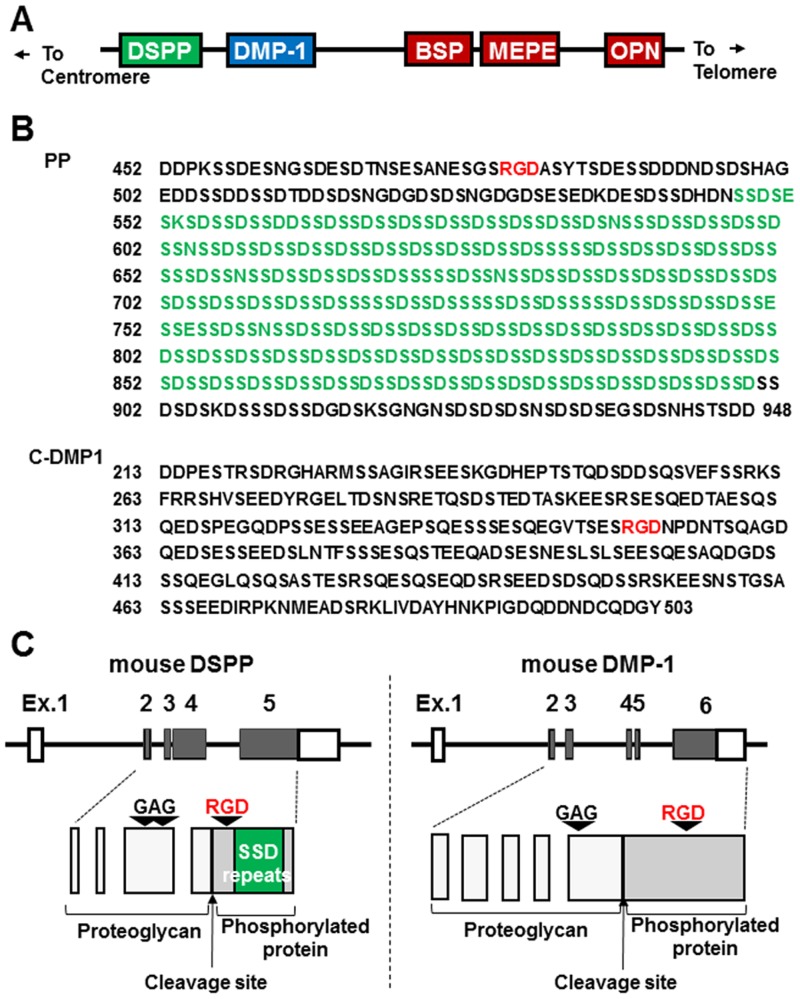
Gene and protein structures of mouse DSPP and DMP-1. (A) SIBLING location. SIBLING proteins were located next to each other on human chromosome 4 and mouse chromosome 5. (B) The amino acids sequences of PP (the carboxyl-terminal region of DSPP) and C-DMP1 (the carboxyl-terminal region of DMP-1). The SSD repeats are colored green and the RGD domains are colored red. The amino acids are numbered from the amino-terminal of DSPP and DMP-1 after signal peptide removal. (C) The exon-intron and protein structures of mouse DSPP and DMP-1. Two glycosaminoglycans (GAG) sites have been reported in mouse DSPP (serine242 and serine254) and one GAG site in DMP1 (serine 89). SSD repeats; Serine–serine–aspartic acid repeat sequence. RGD; Arg-Gly-Asp integrin-binding site.

The small integrin-binding ligand *N*-linked glycoproteins (SIBLINGs) family consists of five extracellular matrix proteins: dentin sialophosphoprotein (DSPP), dentin matrix protein-1 (DMP-1), bone sialoprotein (BSP), matrix extracellular glycophosphoprotein (MEPE), and osteopontin (OPN). Human chromosome 4 (mouse chromosome 5) has been shown to contain a SIBLING family gene cluster (4q21) located immediately proximal to a cluster of enamel matrix protein genes (4q13) (Fig. 1A) [1]–[4]. The SIBLING family has been defined on the basis of the common structural, biochemical, and genetic features of its members [2]. SIBLINGs contain an Arg-Gly-Asp (RGD) integrin-binding site and, thus, are involved in various cellular responses such as migration, differentiation, adhesion, and metastasis [4].

Phosphophoryn (PP) (alternatively referred to as dentin phosphoprotein or dentin phosphophoryn), which is the carboxyl-terminal cleaved product of DSPP, contains the RGD domain and repeat sequences of Ser-Ser-Asp (SSD) (there are over 200 tandem copies in humans and approximately 100 copies in mice) (Fig. 1B). Most SSD repeats are phosphorylated and may be some of the most acidic proteins in the human body [5], [6]. The *Dspp* gene is known to be primarily expressed in odontoblasts and, to a lesser extent, in osteoblasts [7], [8]. *Dspp* is also expressed in other tissues such as the salivary glands, lungs, and kidneys [9]–[11]. Functional analyses in genetically altered mouse models mainly elucidated the function of DSPP as an inducer of mineralization in the extracellular matrix [12]–[14]. An *in vitro* overexpression study revealed that PP induced mineral nodule formation, even in NIH3T3 fibroblast cells [15].

DMP-1 was found to be the most similar to DSPP among the SIBLING members, and these share many similarities in both their gene and protein structures and play important roles in the development of hard tissue (Fig. 1C) [12], [16]–[22]. A previous study indicated that *DSPP* was created due to gene duplications in the ancestor *DMP-1* genomic sequence of toothed animals [23]. DSPP and DMP-1 are both cleaved into two protein chains; the N-terminal regions are proteoglycans that contain chondroitin sulfate chains, and the C-terminal regions are highly phosphorylated. As shown in Figure 1C, PP and carboxyl-terminal DMP-1 (C-DMP-1) both contain the integrin binding site RGD, which is colored red, while PP also includes long SSD repeats, which are colored green. DMP-1 was previously shown to aid adhesion to various cells through integrin receptors [24]. Bone morphogenetic protein 1 (BMP-1) and its alternatively spliced isoform, tolloid (TLD) are known to cleave full-length DMP-1 and DSPP proteins into two proteins [25]–[28].

Yamakoshi *et al.* recently proposed that DSPP should be classified into intrinsically disordered proteins (IDPs) due to its high net charge and low hydrophobicity [29], [30]. IDPs generally do not adopt a defined three-dimensional structure, but, nevertheless, possess important functions *in vivo* and *in vitro* [31]. Since IDPs are known to be highly susceptible to bacterial proteases due to their flexible unfolded structures [32], the mammalian expression system is considered to be better for generating recombinant PP proteins. However, Marschall *et al.* reported that only small amounts of PP-related proteins were secreted from transfected mammalian cells due to their extremely acidic nature and SSD repeats [25]; therefore, the purification of recombinant PP proteins by a mammalian expression system was considered to be difficult.

In the present study, we successfully generated recombinant PP using a mammalian expression system and evaluated its integrin-mediated adhesive effects by simultaneously analyzing the effects of recombinant C-DMP-1 and the well-known integrin ligand vitronectin. Wells coated with recombinant PP did not facilitate cell adhesion, whereas recombinant C-DMP-1 and vitronectin did. Further analyses utilizing various recombinant proteins and peptides containing PP-RGD indicated that the Ala-Ser site flanking the RGD domain was a key peptide bond that allowed the PP-RGD domain to be sequestered.

## Results

### Generation of a rabbit anti-PP antibody and recombinant PP (rPP) protein

**Figure 2:**
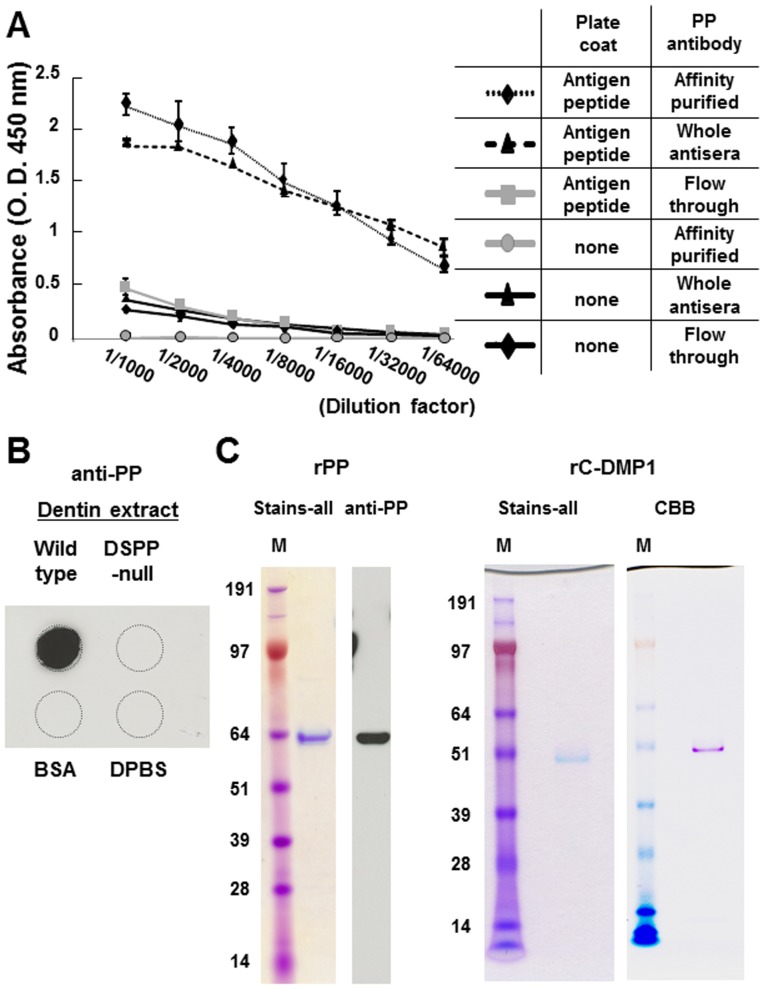
Generation of the affinity-purified rabbit anti-PP antibody and purification of rPP and rC-DMP-1 proteins. (A) The affinity-purified rabbit anti-PP antibody, whole antisera (unpurified anti-PP antisera), and column flow-through solution were titrated using ELISA through serial dilutions ranging from 1∶1,000 to 1∶64,000. Each value represents the mean of triplicate determinations. (B) To examine the specificity of this rabbit anti-PP antibody, the dentin extract was dot-blotted onto a nitrocellulose membrane and PP was detected with the rabbit anti-PP antibody (1∶2,500). (C) The rPP protein (500 ng) appeared as a single band on SDS-PAGE, as observed with Stains-All staining, and the molecular size of this single band was concomitant with the protein band identified by anti-PP. The rC-DMP-1 protein (1 µg) appeared as a single band on SDS-PAGE, as observed using Stains-All and Coomassie brilliant (CBB) staining, which confirmed the purities of these rPP and rC-DMP-1 proteins for further experimental use. Lane M, molecular weight marker.

We first generated an affinity-purified rabbit anti-PP polyclonal antibody to detect rPP. The carboxyl-terminal amino acid sequences of PP (DSEGSDSNHSTSDD) were selected as the antigen peptide based on low sequence similarities. The rabbit anti-PP antibody was generated by serial vaccinations using the antigen peptide as described. To examine the antigen recognition capacity of the rabbit anti-PP antibody, the affinity-purified rabbit anti-PP antibody, whole antisera (unpurified anti-PP antisera), and column flow-through solution were titrated using an enzyme-linked immunosorbent assay (ELISA) with serial dilutions ranging from 1∶1,000 to 1∶64,000. Relative to the column flow-through solution, the affinity-purified rabbit anti-PP antibody as well as whole antisera showed a positive dilution from 1∶64,000, which indicated that the rabbit anti-PP antibody retained its antigen recognition ability following affinity purification (Fig. 2A). To examine the specificity of this rabbit anti-PP antibody, a dentin extract was dot-blotted onto a nitrocellulose membrane and PP was detected with the rabbit anti-PP antibody. The rabbit anti-PP antibody only reacted with the dentin extract of wild type mice and not with that of DSPP-null mice. The rabbit anti-PP antibody reacted with neither bovine serum albumin (BSA) nor Dulbecco's Phosphate-Buffered Saline (DPBS) (Fig. 2B).

As shown in Figure 2C, rPP appeared as a single band that migrated to 60 kDa on SDS-PAGE, as observed using Stains-All staining, and the molecular size of this single band was concomitant with the protein band identified by the anti-PP antibody and anti-6xHis antibody (data not shown). rC-DMP-1 proteins appeared as a single band that migrated to 50 kDa on SDS-PAGE, as observed using Stains-All and Coomassie brilliant staining, and the molecular size of this single band was consistent with the protein band identified by the anti-6xHis antibody (data not shown). These results confirmed that the purities of rPP and rC-DMP-1 were suitable for further experimental use.

### Differential cell adhesive abilities of rPP, rC-DMP-1, and vitronectin

**Figure 3:**
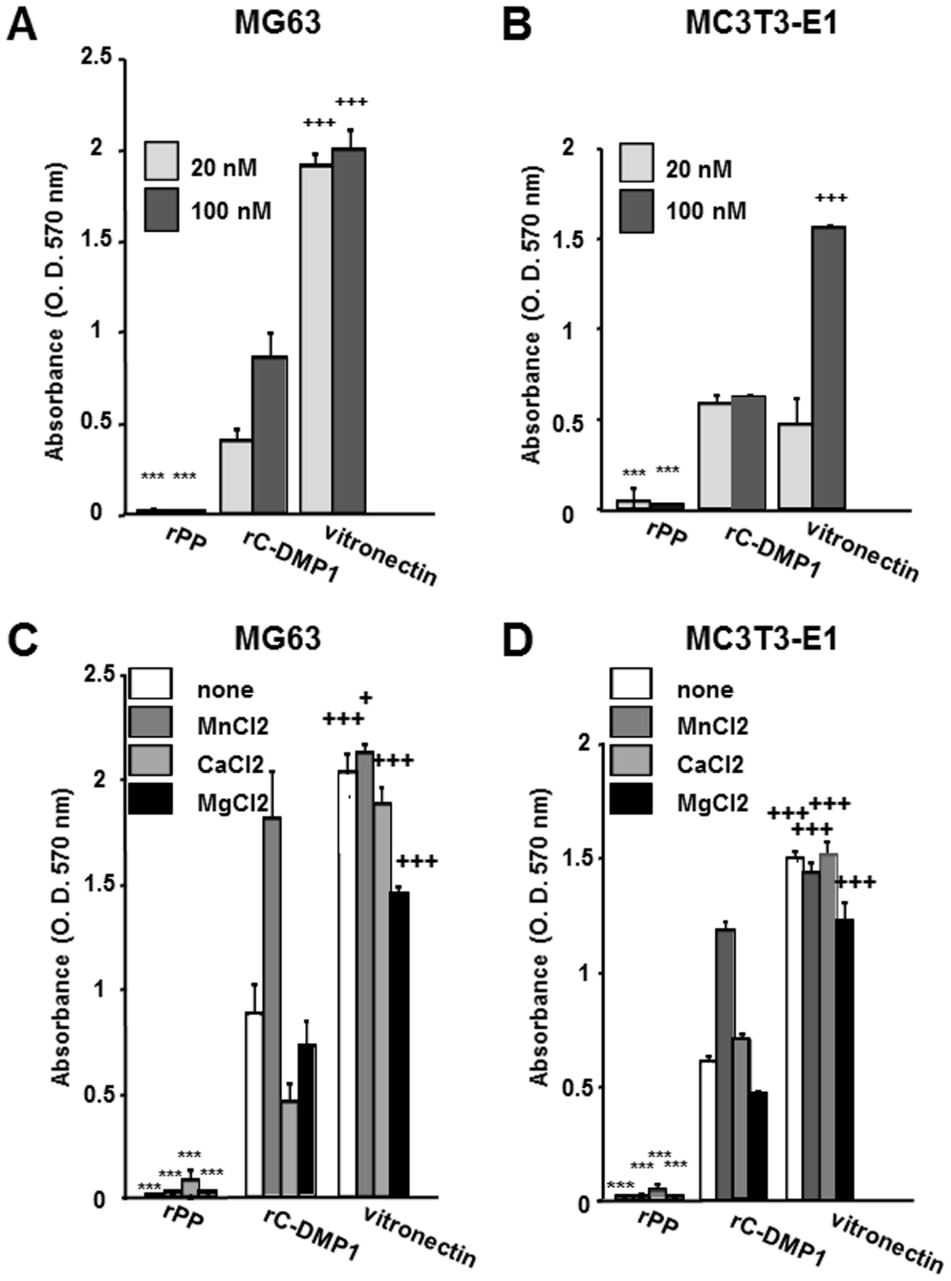
Nominal effects of rPP on MG63 and MC3T3-E1 cell adhesion. Ninety-six-well plates were precoated with 20 or 100 nM rPP, rC-DMP-1, and vitronectin, seeded with MG63 (A) and MC3T3-E1 (B) cells in serum-free medium, and incubated for 1 hr. MG63 (C) and MC3T3-E1 (D) cells were seeded onto 100 nM rPP, rC-DMP-1, and vitronectin with either MnCl_2_, CaCl_2_, or MgCl_2_ (1 mM) in serum-free medium, and incubated for 1 hr. After washing non-adherent cells, the attached cells were stained with 0.2% crystal violet and dissolved in 1% SDS solution. Absorbance was measured at 570 nm. Each value represents the mean of triplicate determinations; bars mean ±SD. Statistical analysis was performed by a one-way ANOVA, followed by Dunnett's test. ***p<0.001 indicates significantly lower and ^+^p<0.05 and ^+++^p<0.001 indicate significantly higher than rC-DMP-1-coated wells at the same concentration (A and B) or rC-DMP-1-coated wells with the same divalent cation (C and D).

Utilizing MG63 and MC3T3-E1 cells (unless otherwise noted, MC3T3-E1 cells refer to subclone 4 in this study), the cell adhesive ability of rPP was simultaneously examined with rC-DMP-1 and vitronectin. These cells were unable to attach to wells coated with 20 or 100 nM rPP, but did attach to wells coated with 20 or 100 nM rC-DMP-1 and vitronectin. The adhesive potency of these cells was more profound on vitronectin than on rC-DMP-1 (Fig. 3A and B). We then evaluated the effects of MnCl_2_, CaCl_2_, and MgCl_2_ on cell adhesion to these proteins. As shown in Figure 3C and D, none of the divalent cations enhanced the cell adhesion of MG63 and MC3T3-E1 cells to rPP. In contrast, the addition of MnCl_2_ strongly potentiated cell adhesion to rC-DMP-1.

### Six other cells were also unable to attach to rPP

**Figure 4:**
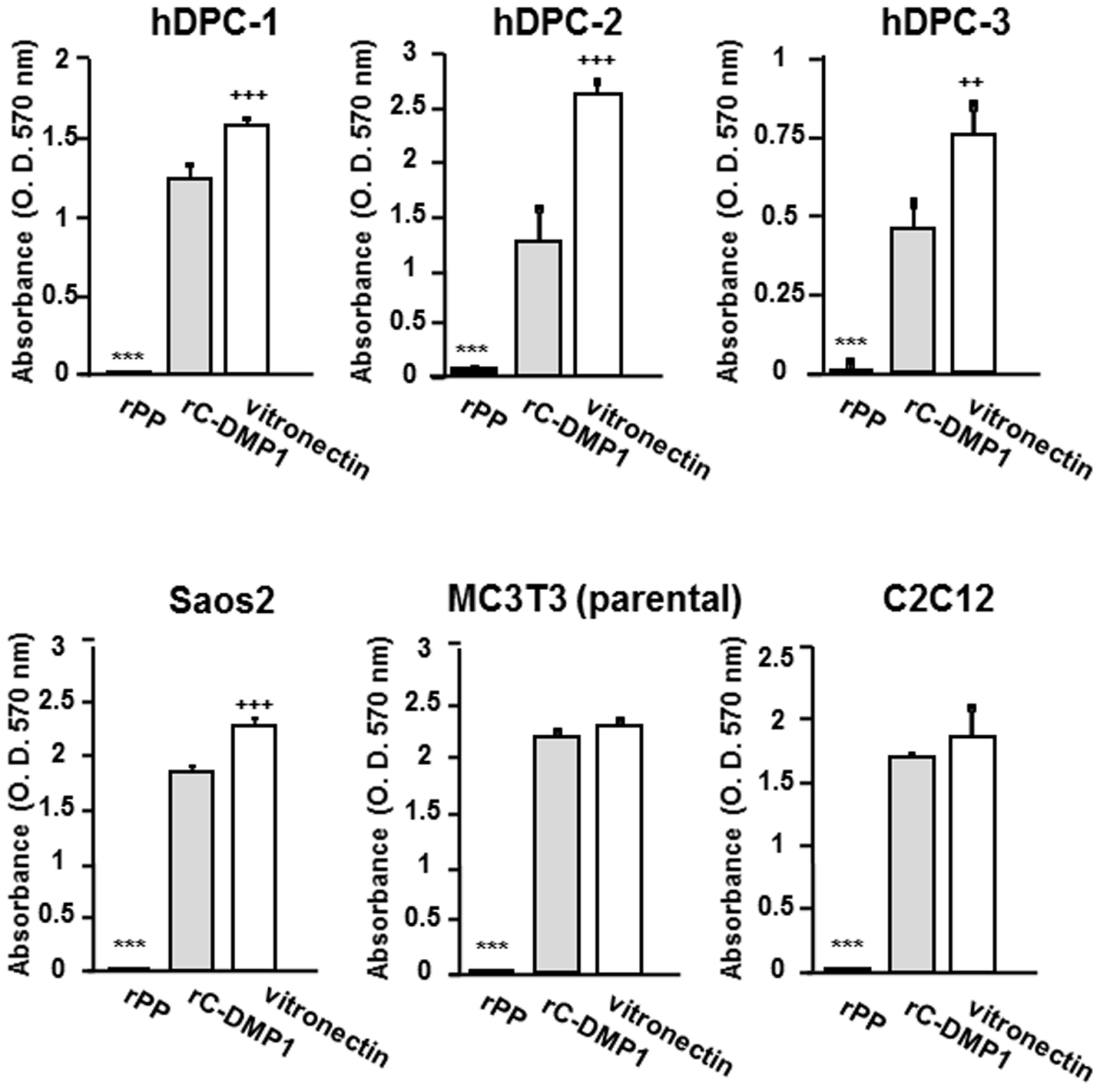
Six other mesenchymal cell lines were unable to attach to rPP. Three hDPC (human dental pulp cells) cells from different donors, Saos2 (human osteosarcoma cell line), parental MC3T3-E1 (heterogeneous cell population), and C2C12 (mouse myoblast cell line) were seeded onto rPP, rC-DMP-1, and vitronectin (100 nM) with MnCl_2_ (1 mM). The number of attached cells was evaluated as described before. Each value represents the mean of triplicate determinations; bars mean ±SD. Statistical analysis was performed by a one-way ANOVA, followed by Dunnett's test. ***p<0.001 indicates significantly lower and ^++^p<0.01 and ^+++^p<0.001 indicate significantly higher than rC-DMP-1-coated wells.

Since rPP was incapable of aiding cell adhesion to human osteosarcoma MG63 cells or mouse pre-osteoblastic MC3T3-E1 cells, we examined various cell adhesions on rPP in parallel with rC-DMP-1 and vitronectin with the addition of MnCl_2_. As shown in Figure 4, three human dental pulp (hDPC) cells, the human osteosarcoma cell line, SaoS2, parental heterogeneous MC3T3-E1 cells, and mouse myoblast cell line, C2C12 were clearly unable to attach to wells coated with 100 nM rPP, but did attach to wells coated with 100 nM rC-DMP-1 and vitronectin, which was similar to the results obtained for MG63 and MC3T3-E1 cells.

### The removal of SSD repeats in PP had no apparent influence on its adhesive potency

**Figure 5:**
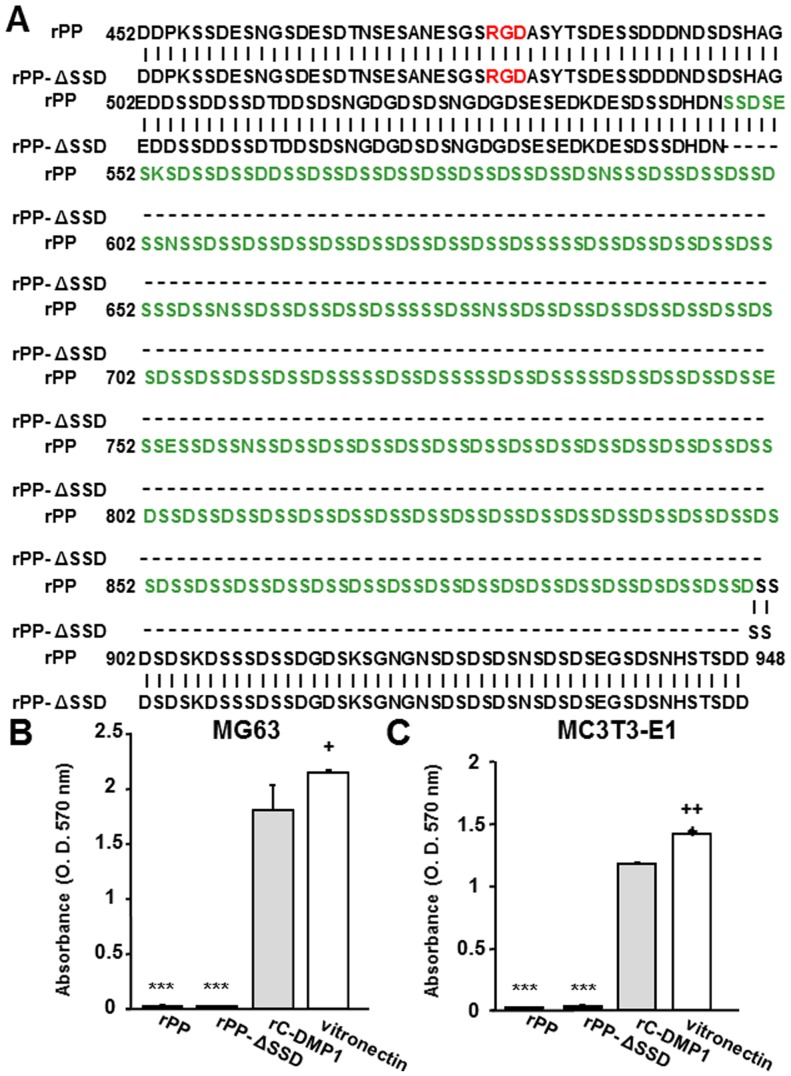
The removal of SSD repeats had no apparent effect on the adhesive ability of rPP. (A) Comparative deduced amino acid sequences of rPP and rPP-ΔSSD. The RGD domain is colored red. The amino acid sequences excluded in rPP-ΔSSD are colored green. MG63 (B) and MC3T3-E1 (C) cells were seeded onto rPP, rPP-ΔSSD, rC-DMP-1, and vitronectin (100 nM) with MnCl_2_ (1 mM). The number of attached cells was evaluated as described above. Each value represents the mean of triplicate determinations; bars mean ±SD. Statistical analysis was performed by a one-way ANOVA, followed by Dunnett's test. ***p <0.001 indicates significantly lower and ^+^p<0.05 and ^++^p<0.01 indicate significantly higher than rC-DMP-1-coated wells.

The most characteristic feature of PP is the SSD repeat in its central portion. Therefore, we speculated that SSD repeats may somehow negatively affect cell adhesion to mask the RGD-dependent adhesive potency of rPP. To examine this hypothesis, we generated rPP-ΔSSD in which the SSD repeats were removed (the deduced amino acid sequences of rPP and rPP-ΔSSD were shown in Fig. 5A). As shown in Figure 5B and C, MG63 and MC3T3-E1 cells were unable to attach to PP-ΔSSD, which was consistent with the results obtained for rPP. These results indicated that the lack of binding potency by rPP could not be attributed to the SSD repeats.

### rC-DMP-1 and vitronectin, but not rPP directly associated with integrin αvβ3 and αvβ5

**Figure 6:**
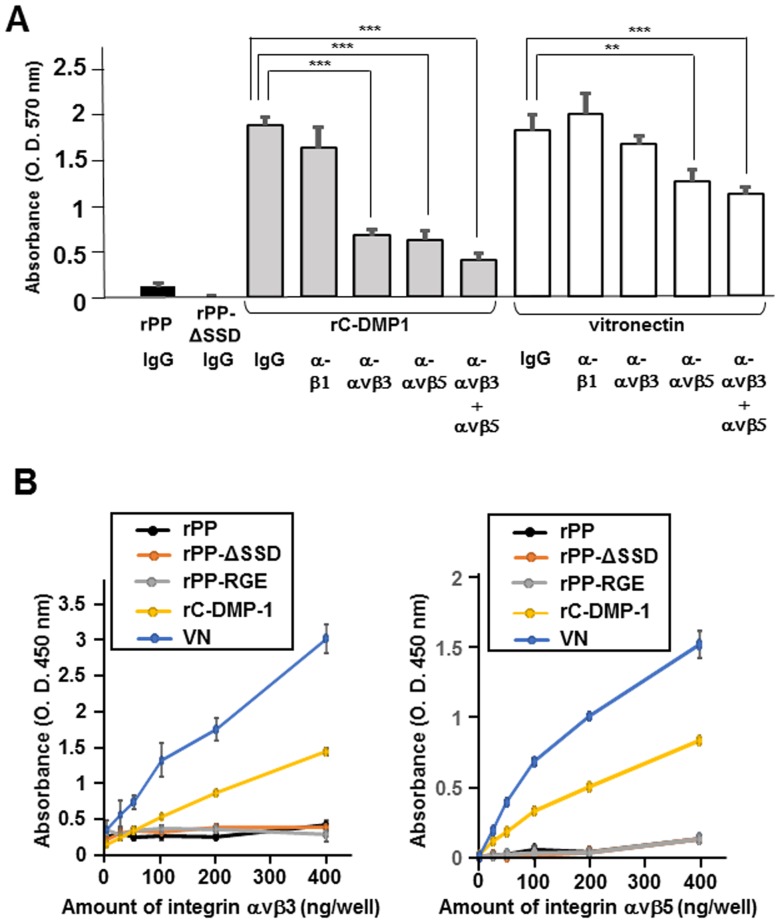
The inability of rPP, rPP-ΔSSD, and rPP-RGE to bind to integrin αvβ3 or αvβ5. (A) MG63 cells were preincubated with the neutralizing antibodies against integrin αvβ3 (LM609), αvβ5 (P1F6), and β1 (4B7) and control IgG (10 µg/ml) for 15 min, and were then seeded onto rC-DMP-1 and vitronectin (100 nM) in serum-free medium. Statistical analysis was performed by a one-way ANOVA, followed by Dunnett's test. **p<0.01 and ***p<0.001 indicate significantly lower than the cells preincubated with control IgG. (B) The binding of integrin αvβ3 and αvβ5 to rPP, rPP-ΔSSD, rPP-RGE, rC-DMP-1, and vitronectin was analyzed using solid phase binding assays. Various amounts (0∼400 ng) of integrin αvβ3 and αvβ5 were added to 96-well plates precoated with either 100 nM of PP, PP-ΔSSD, PP-RGE, rC-DMP-1, or vitronectin. Bound integrin αvβ3 and αvβ5 were then detected with the anti-integrin αv antibody, an appropriate HRP-conjugated secondary antibody, and TMB. Each value represents the mean of triplicate determinations; bars mean ±SD.

Since rC-DMP-1 and vitronectin were able to support MG63 cell adhesion, we pre-incubated these cells with neutralizing antibodies against human integrin β1, αvβ3, and αvβ5 before seeding onto rC-DMP-1 and vitronectin (Fig. 6A). The number of cells that attached to rC-DMP-1 was significantly lower following the preincubation with antibodies against integrin αvβ3 and αvβ5 than that with control IgG. Moreover, the co-addition of neutralizing antibodies against αvβ3 and αvβ5 more potently reduced the number of attached cells. The number of cells that attached to vitronectin was profoundly inhibited by the preincubation with the neutralizing antibody against integrin αvβ5 and was slightly inhibited by the preincubation with the neutralizing antibody against integrin αvβ3. The preincubation with the neutralizing antibody against integrin β1 was unable to inhibit MG63 cell adhesion to either rC-DMP-1 or vitronectin. Therefore, we hypothesized that the inability of rPP to facilitate MG63 cell adhesion may be attributed to the inability of rPP to associate with integrin αvβ3 and αvβ5. We examined the binding abilities of rPP, rPP-ΔSSD, rPP-RGE, which was the RGD-inactivated mutant of rPP, rC-DMP-1, and vitronectin to integrin αvβ3 and αvβ5. As shown in Figure 6B, integrin αvβ3 and αvβ5 were able to associate with rC-DMP-1 and vitronectin in dose-dependent manners, with the binding capability of vitronectin being higher than that of rC-DMP-1. However, integrin αvβ3 or αvβ5 did not bind to rPP, rPP-ΔSSD, or rPP-RGE. This result demonstrated that the adhesive inability of rPP was presumably due to its inability to bind to the integrin receptors.

### Identification of flanking amino acid sequences to allow the RGD domain to become inactive

**Figure 7:**
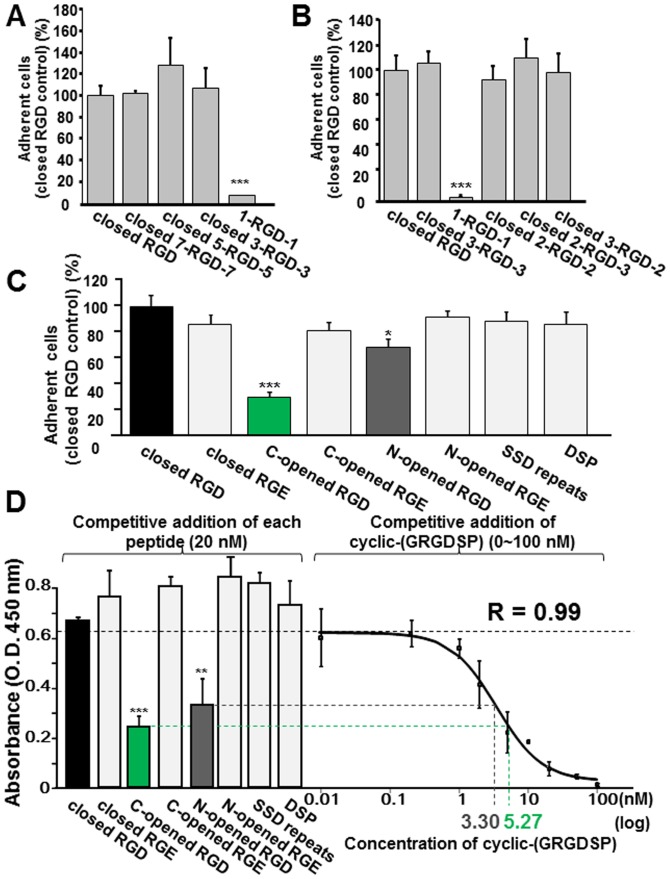
Ala^482^-Ser^483^ was the key peptide bond that allowed the PP-RGD domain to become inactive. (A) MG63 cells were preincubated with closed RGD, closed 7-RGD-7, closed 5-RGD-5, closed 3-RGD-3, and 1-RGD-1 peptides (1 mM) for 10 min, and were then seeded onto vitronectin (100 nM) in serum-free medium. (B) The minimum amino acid sequences needed to sequester the ability of PP-RGD were narrowed down based on the results of (A). MG63 cells were preincubated with closed RGD, closed 3-RGD-3, 1-RGD-1, closed 2-RGD-2, closed 2-RGD-3, and closed 3-RGD-2 peptides (1 mM). (C) The various PP peptides exhibited different inhibitory activities on cell adhesion to vitronectin. MG63 cells were preincubated with the various PP peptides: closed RGD, closed RGE, C-opened RGD, C-opened RGE, N-opened RGD, N-opened RGE, SSD repeats, or DSP (1 mM) as described, and were then seeded onto vitronectin. C-opened RGD significantly inhibited the adhesion of these cells to vitronectin. The number of attached cells was evaluated as described above. Cell attachment in the presence of closed RGD was assigned a value of 100%. Each value represents the mean of triplicate determinations; bars mean ±SD. Statistical analysis was performed by a one-way ANOVA, followed by Tukey's test. *p<0.05 and ***p<0.001 significantly different from the cells preincubated with any other peptide. (D) The various PP peptides showed different binding capacities to integrin αvβ3. The biotinylated GRGDS peptide (5 nM) was added to integrin αvβ3-coated wells with various PP peptides or with serially-diluted cyclic-(GRGDP). The bound biotinylated GRGDS peptide was valued using a streptavidin-HRP conjugated polymer. Dose-dependent decreases in biotinylated GRGDS binding to integrin αvβ3 were regressed to a sigmoid curve. The inhibitory values of the various PP peptides were determined by converting their absorbance to the inhibitory value of cyclic-(GRGDSP). Each value represents the mean of triplicate determinations; bars, mean ±SD. Statistical analysis was performed by a one-way ANOVA, followed by Dunnett's test. **p<0.01 and ***p<0.001 indicate significantly lower than the competitive addition of closed RGD.

We hypothesized that a specific flanking amino acid sequence must exist in the vicinity of the RGD domain to sequester the function of PP-RGD. Therefore, we generated various kinds of mouse PP peptides, as shown in Table 1: H-SESANESGSRGDASYTSDESS-OH (closed RGD), H-SANESGSRGDASYTSDE-OH (closed 7-RGD-7), H-NESGSRGDASYTS-OH (closed 5-RGD-5), H-SGSRGDASY-OH (closed 3-RGD-3), and H-SRGDA-OH (1-RGD-1). These peptides contained the RGD domain at their centers and 9, 7, 5, 3, and 1 juxta-amino acids were attached at both the amino- and carboxyl-terminal sides. We preincubated MG63 cells with these peptides before seeding onto vitronectin and then examined whether these peptides were able to inhibit the adhesion of MG63 cells to vitronectin. As shown in Figure 7A, MG63 cell adhesion to vitronectin was significantly lower when these cells were preincubated with 1-RGD-1 than with closed RGD, closed 7-RGD-7, closed 5-RGD, and closed 3-RGD-3. As shown in Figure 7B, we then generated a peptide with 2 amino acids at both sides of RGD (closed 2-RGD-2) (Table 1) and found that the preincubation of MG63 cells with this peptide was unable to inhibit binding to vitronectin, which was also observed with closed 3-RGD-3. This result was supported by the same inability of the preincubation with peptides having two and three (closed 2-RGD-3) (Table 1), and three and two (closed 3-RGD-2) (Table 1) amino acids at the amino- and carboxyl-terminals of RGD, respectively, to inhibit binding to vitronectin.

**Table 1:** The PP-delivered peptide list.

closed RGD	SESANESGS**RGD**ASYTSDESS
closed 7-RGD-7	SANESGS**RGD**ASYTSDE
closed 5-RGD-5	NESGS**RGD**ASYTS
closed 3-RGD-3	SGS**RGD**ASY
1-RGD-1	S**RGD**A
closed 2-RGD-2	GS**RGD**AS
closed 2-RGD-3	GS**RGD**ASY
closed 3-RGD-2	SGS**RGD**AS
closed RGE	SESANESGSRGEASYTSDESS
C-opened RGD	SDESDTNSESANESGS**RGD**A
C-opened RGE	SDESDTNSESANESGSRGEA
N-opened RGD	S**RGD**ASYTSDESSDDDNDSDSH
N-opened RGE	SRGEASYTSDESSDDDNDSDSH
SSD repeats	DSSDSSDSSDSSDSSNSS
DSP	PSGNGVEEDEDTGSGDGE

Closed RGD, closed 7-RGD-7, closed 5-RGD-5, closed 3-RGD-3, and 1-RGD-1 contained 9, 7, 5, 3, and 1 juxta-amino acids at the amino- and carboxyl-terminal sides of RGD, respectively. Closed 2-RGD-2 had 2 amino acids at both sides of RGD. Closed 2-RGD-3 had two and three, and closed 3-RGD-2 had three and two amino acids at the amino- and carboxyl-terminal sides of RGD. Close RGE was the RGD-inactivated mutant of closed RGD. C-opened RGD included the RGD domain with an open carboxyl-terminal side, and C-opened RGE was the RGD-inactivated mutant of C-opened RGD. N-opened RGD included the RGD domain with an open amino-terminal side and N-opened RGE was the RGD-inactivated mutant of N-opened RGD. SSD repeats had representative SSD repeats. DSP was the well-conserved glycosaminoglycan attachment site in the dentin sialoprotein (DSP), which was the amino-terminal cleaved product of DSPP.

We demonstrated that the incorporation of more than one amino acid sequestered the ability of the RGD domain. However, it remains unknown whether the incorporation of amino acids at both the amino- and carboxyl-terminal sides or at either one of these sides was sufficient to allow the PP-RGD domain to become inactive. Thus, we newly synthesized various kinds of PP peptides as shown in Table 1. H-SESANESGSRGEASYTSDESS-OH (closed RGE) was the RGD-inactivated mutant of closed RGD. H-SDESDTNSESANESGSRGDA-OH (C-opened RGD) included the RGD domain with an open carboxyl-terminal side, and H-SDESDTNSESANESGSRGEA-OH (C-opened RGE) was the RGD-inactivated mutant of C-opened RGD. H-SRGDASYTSDESSDDDNDSDSH-OH (N-opened RGD) included the RGD domain with an open amino-terminal side and H-SRGEASYTSDESSDDDNDSDSH-OH (N-opened RGE) was the RGD-inactivated mutant of N-opened RGD. H-DSSDSSDSSDSSDSSNSS-OH (SSD repeats) had the representative SSD repeats. H-PSGNGVEEDEDTGSGDGE-OH (DSP) was the well-conserved glycosaminoglycan attachment site in the dentin sialoprotein (DSP), which is the amino-terminal cleaved product of DSPP. As described above, we preincubated MG63 cells with these peptides and evaluated their RGD domain activities by analyzing their inhibitory effects on the adhesion of MG63 cells to vitronectin. The adhesion of MG63 cells was significantly inhibited by the preincubation with C-opened RGD, but not by that with its RGD-inactivated mutant, C-opened RGE. The preincubation with N-opened RGD slightly inhibited cell adhesion; however, its effect was not as marked as that of C-opened RGD. As expected, closed RGE and N-opened RGE did not alter the number of attached cells. SSD repeats and the DSP peptide did not affect the adhesion of MG63 cells to vitronectin.

We then performed a competitive integrin binding assay. As shown in Figure 7D, among closed RGD, closed RGE, C-opened RGD, C-opened RGE, N-opened RGD, N-opened RGE, SSD repeats, and the DSP peptides, the competitive addition of C-opened RGD or N-opened RGD (20 nM) significantly reduced biotinylated GRGDS (5 nM) binding to integrin αvβ3. To determine the binding potencies of these peptides, we used serially-diluted cyclic-(GRGDSP) to compete with biotinylated GRGDS. Dose-dependent inhibition by cyclic-(GRGDSP) was regressed to a sigmoid curve (R = 0.99) and the inhibitory values of these peptides were determined by converting their absorbances to the inhibitory value of cyclic-(GRGDSP). The activities of 20 nM C-opened RGD and N-opened RGD were equal to 5.27 (26.5%) and 3.30 (16.5%) nM of cyclic-(GRGDSP), respectively. The activity of the same amount of cyclic-(GRGDSP) (20 nM) was assigned a value of 100%. Since neither closed RGD, closed RGE, C-opened RGE, N-opened RGE, SSD repeats, nor the DSP peptide exhibited inhibitory effects, their converted values were not determined.

### Cell adhesion and migration to rPP with an exposed carboxyl-terminal side of the RGD domain

**Figure 8:**
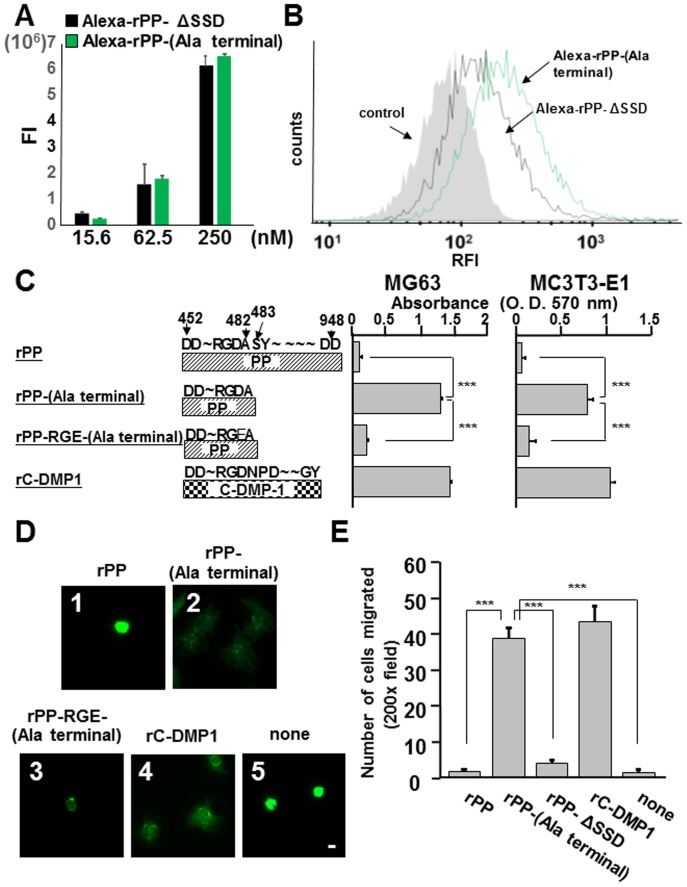
Truncated rPP terminating with Ala^482^ (PP-(Ala terminal)) induced cell adhesion and migration. (A) The fluorescence intensity (FI) of Alexa-Fluor 488 conjugated rPP-ΔSSD and rPP-(Ala terminal). One hundred microliters of serially-diluted Alexa-Fluor 488 conjugated rPP-ΔSSD (Alexa-rPP-ΔSSD) and rPP-(Ala terminal) (Alexa-rPP-(Ala terminal)) were added onto black 96-well plates and FI was measured on a fluorescence microplate reader. Their FI values were equivalent. (B) Flow cytometry analysis of Alexa-rPP-ΔSSD and Alexa-rPP-(Ala terminal) binding to MG63 cells. MG63 cells were incubated with Alexa-rPP-ΔSSD and Alexa-rPP-(Ala terminal) (250 nM). rPP-(Ala terminal) binding to MG63 cells was apparently higher than that of rPP-ΔSSD and control cells, which were incubated with medium only. (C) MG63 and MC3T3-E1 cells were seeded onto rPP, rPP-(Ala terminal), rPP-RGE-(Ala terminal), and rC-DMP-1 (100 nM) with MnCl_2_ (1 mM) in serum-free medium. The number of attached cells was evaluated as described above. Each value represents the mean of triplicate determinations; bars mean ±SD. (D) Organization of actin stress fibers on rPP, rPP-(Ala terminal), rPP-RGE-(Ala terminal), and rC-DMP-1. MG63 cells were seeded onto glass plates precoated with rPP (box 1), rPP-(Ala terminal) (2), rPP-RGE-(Ala terminal) (3), rC-DMP-1 (4), or none (5) (100 nM) in serum-free medium. Actin fibers were visualized by FITC-phalloidin 1 hr after seeding. Bar  = 10 µm. (E) rPP-(Ala terminal) was able to induce haptotaxis cell migration. Haptotaxis cell migration was measured in MG63 cells toward rPP, rPP-(Ala terminal), rPP-ΔSSD, and rC-DMP-1 in serum-free medium. After the 12-hr incubation, cells were fixed and stained. Migration was quantified by counting the number of cells that migrated through filters. Each value represents the mean of 10 randomly selected fields at 200× magnification; bars mean ±SD. Statistical analysis was performed by a one-way ANOVA, followed by Dunnett's test. ***p<0.001 significantly lower than PP-(A terminal)-coated wells.

Since the carboxyl-terminal side of RGD was considered to be more essential than the amino-terminal side in sequestration of the ability of PP-RGD, we generated a hypothetical truncated rPP fragment (rPP-(Ala terminal) protein) that terminated with Ala^482^ next to the RGD domain and compared the binding abilities of rPP-ΔSSD and rPP-(Ala terminal) to MG63 cells by flow cytometry. We mixed rPP-ΔSSD and rPP-(Ala terminal) with the Alexa Fluor 488 tetrafluorophenyl ester at the same dye: protein molar ratio (30: 1) for conjugation and their equivalent labeling degrees were validated by measuring their fluorescence intensities (FI) in 96-well plates (Fig. 8A). As shown in Figure 8B, MG63 cells incubated with Alexa-rPP-(Ala terminal) had an apparently higher FI than those incubated with Alexa-rPP-ΔSSD and the control cells. Cells incubated with Alexa-rPP-ΔSSD showed a slightly higher FI than that of the control cells. We then analyzed the cell-adhesive ability of rPP-(Ala terminal) and found that the abilities of MG63 and MC3T3-E1 cells to attach to rPP-(Ala terminal)-coated wells were similar to their abilities to attach to rC-DMP-1-coated wells (Fig. 8C column 2 and 4). On the other hand, MG63 and MC3T3-E1 cells were unable to attach to rPP-RGE-(Ala terminal)-coated wells (column 3), which was the RGD-inactivated mutant of rPP-(Ala terminal), or to rPP-coated wells (column 1). Moreover, MG63 cells spread and formed actin stress fibers on rPP-(Ala terminal)-coated wells as well as rC-DMP1-coated wells (Fig. 8D, boxes 2 and 4). However, cell adhesion and organization were delayed and cells remained round on rPP (box 1), rPP-RGE-(Ala terminal) (box 3), and non-coated wells (box 5). The organization of the actin stress fibers of MC3T3-E1 cells was similar on these substrates (data not shown).

Cell matrix interactions between RGD domains and integrin receptors commonly mediate cell migration as well as cell adhesion. Therefore, we examined the effects of rPP, rPP-(Ala terminal), rPP-ΔSSD, and rC-DMP1 proteins on haptotaxis cell migration by the modified Boyden chamber assay using the Transwell system. We coated the bottom side of the membrane with rPP, rPP-(Ala terminal), rPP-ΔSSD, or rC-DMP1 and counted the number of MG63 cells that migrated from the upper side to the bottom side of the membrane. We found that rPP-(Ala terminal) and rC-DMP1 were able to induce haptotaxis migration by MG63 cells, whereas rPP and rPP-ΔSSD were not (Fig. 8E).

### The Ala-Ser bond was the key flanking peptide bond that allowed primate and mouse PP-RGD domains to become inactive

**Figure 9:**
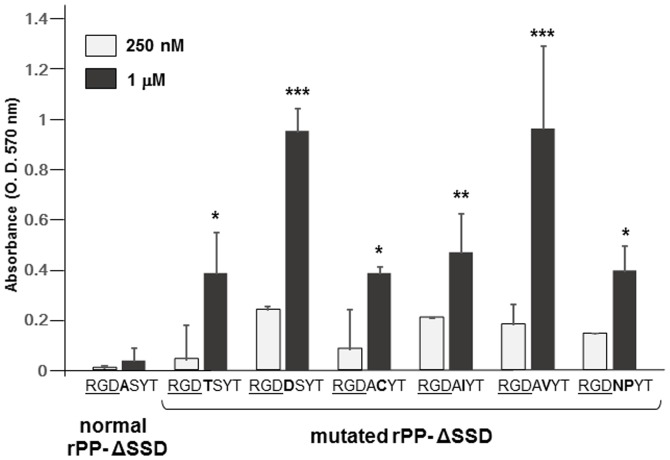
Replacement of the 1^st^ and/or 2^nd^ carboxyl-terminal amino acids of the PP-RGD domain altered its ability. Ninety-six-well plates were precoated with 250 nM or 1 µM of normal rPP-ΔSSD and various mutated rPP-ΔSSD proteins, seeded with MG63 cells in serum-free medium, and incubated for 1 hr. The number of attached cells was evaluated as described above. Each value represents the mean of triplicate determinations; bars mean ±SD. Statistical analysis was performed by a one-way ANOVA, followed by Dunnett's test. *p<0.05, **p<0.01, and ***p<0.001 indicate significantly higher than rPP-ΔSSD-coated wells at the same concentration.

Since the release of Ser^483^ from the vicinity of RGD disclosed PP-RGD ability, we speculated that the Ala-Ser peptide bond may be the key to sequestering the ability of the RGD domain to act on integrin. Therefore, we newly generated rPP-ΔSSD-RGDTSYT in which Ala^482^ was replaced with Thr, rPP-ΔSSD-RGDDSYT in which Ala^482^ was replaced with Asp, rPP-ΔSSD-RGDACYT in which Ser^483^ was replaced with Cys, rPP-ΔSSD-RGDAIYT in which Ser^483^ was replaced with Ile, rPP-ΔSSD-RGDAVYT in which Ser^483^ was replaced with Val, and rPP-ΔSSD-RGDNPYT in which Ala^482^-Ser^483^bond was replaced with an Asn-Pro bond to mimic the human and mouse DMP-1-RGD domains. We then examined their cell-adhesive potencies by quantifying the number of attached MG63 cells on wells coated with normal rPP-ΔSSD and these mutated rPP-ΔSSD proteins. As shown in Figure 9, MG63 cells were able to attach to the plates coated with 1 µM of these mutated rPP-ΔSSD proteins, with rPP-ΔSSD-RGDDSYT and rPP-ΔSSD-RGDAVYT showing higher binding capacities. In contrast, MG63 cells were unable to attach to plates coated with 1 µM of normal rPP-ΔSSD. The binding ability of MG63 cells was higher on plates coated with mutated rPP-ΔSSD proteins than with rPP-ΔSSD at 250 nM; however, the cell-adhesive effects of mutated rPP-ΔSSD proteins were not significant at this concentration.

## Discussion

In the present study, we demonstrated that neither rPP nor rPP-ΔSSD exhibited any adhesive or migratory ability, whereas the simultaneously purified rC-DMP-1 did. Further analyses utilizing various peptides containing the PP-RGD domains revealed that the Ala^482^-Ser^483^ flanking sequence next to the RGD domain was the key peptide bond that allowed the PP-RGD domain to become inactive.

Vitronectin is an extensively examined adhesive glycoprotein that has been purified from the plasma and ligands of integrin αvβ3 and αvβ5 [33]–[39]. We showed that that adhesion of MG63 cells to rC-DMP-1 and vitronectin was mediated by integrin αvβ3 and αvβ5 (Fig. 6). Thus, we investigated the adhesive potency of rPP using the known adhesive proteins, rC-DMP-1 and vitronectin as positive controls. We mainly used the human osteosarcoma cell line MG63, which has been shown to express several integrin receptors of SIBLING members [24]. As shown in Figure 3A and B, rPP did not exhibit any adhesive potency. Since divalent cations such as MnCl_2_, CaCl_2_, and MgCl_2_ have been shown to potentiate RGD-integrin-mediated signaling [40], [41], we seeded MG63 and MC3T3-E1 cells with divalent cations; however, no divalent cation clearly induced cell adhesion to rPP (Fig. 3C and D). A recent study examined the adhesion of C3H/10T1/2 cells to a relatively low amount (750 ng/ml) of rPP [42] (No description regarding the expression system was provided in this study. Bacterial bovine rPP was used in a related study [43]). The cell adhesion assay performed after 4 or 24 hours of incubation may have led to their conclusion. In our study, we performed all cell adhesion assays within a short time frame (1 hr) without serum in order to exclude the possibility that any pro-adhesive proteins secreted from seeded cells unexpectedly aided adhesion to the coated proteins. We also coated wells with various concentrations (1.7∼1333 nM i. e. 0.1∼80 µg/ml) of intact rPP to determine whether MG63 and MC3T3-E1 cells were able to attach to the wells, and found that no concentration of rPP aided cell adhesion (data not shown). We also confirmed that C3H/10T1/2 cells were unable to attach to wells precoated with rPP (100 nM) within a short time frame (1 hr) without serum. Therefore, we speculated that C3H/10T1/2 cells may secrete a certain amount of pro-adhesive proteins to aid their adhesion to coated proteins in the long term.

The inability of rPP to facilitate cell adhesion was observed when not only MG63 and MC3T3-E1 cells, but also six different mesenchymal cell lines were seeded onto rPP (Fig. 4). Therefore, we concluded that the adhesive inability of rPP was universally observed rather than being osteoblastic cell-specific. As shown in Figure 5, rPP-ΔSSD did not have any positive effects on cell adhesion, which was similar to rPP. This result was supported by the SSD representative peptide being unable to inhibit cell adhesion (Fig. 7C). Thus, we concluded that the lack of binding potency was not due to the SSD repeats of rPP, but could be attributed to their inability to bind to integrin receptors (Fig. 6B).

RGD-containing proteins do not always possess the ability to act on integrin because the RGD domain may not be localized on their surfaces [36]. We preheated rPP at 70°C for 20 min before coating onto 96-well plates; however, MG63 cells were still unable to adhere to these wells (data not shown). This result indicated that the inaccessibility of PP-RGD was not due to the three-dimensional structure of rPP. The accessibility of integrin to the RGD domains is also known to be influenced by the flanking amino acid sequences of the RGD domain [36]. Therefore, we hypothesized that the flanking amino acid sequences may be responsible for allowing the PP-RGD domain to be sequestered. An adhesion assay utilizing several peptides narrowed and identified the exact amino acids of PP in order to allow the PP-RGD domain to become inactive (Fig. 7). The inhibitory ability of 20 nM of the C-opened RGD peptide (having 5.27 nM of the binding activity of cyclic-(GRGDSP) to integrin αvβ3) was more potent than that of 20 nM of the N-opened RGD peptide (having 3.30 nM of the binding activity of cyclic-(GRGDSP) to integrin αvβ3) (Fig. 7D). Therefore, we generated the hypothetical recombinant amino-terminal products of PP, which terminated at Ala^482^ next to the RGD domain, and demonstrated that Alexa-rPP-(Ala terminal) was able to bind to MG63 cells using flow cytometry. MG63 cells incubated with Alexa-rPP-ΔSSD also had a higher FI than control cells. A recent study reported that the highly acidic carboxyl-terminal of rat PP could be endocytosized into different mesenchymal cells even at 4°C for 15 min [44]. Since rPP-ΔSSD includes part of the corresponding amino acid sequence, the slight increase in FI in MG63 cells incubated with Alexa-rPP-ΔSSD could be attributed to this unique endocytic mechanism. We then demonstrated the RGD-dependent effects of rPP-(Ala terminal) on cell adhesion and migration (Fig. 8). These results suggested that the Ala-Ser bond next to the RGD domain was indispensable for sequestering the activity of PP-RGD.

Previous studies showed that the exogenous addition of the porcine PP-RGD peptide, which had Thr next to the RGD domain, induced the cellular migration of human dental pulp cells [45]. Therefore, we determined whether point mutations in Ala and/or Ser altered the ability of the PP-RGD domain (Fig. 9). We examined 6 kinds of mutations and found that all converted rPP-ΔSSD into a cell-adhesive protein. We compared the cell-adhesive abilities of rPP-ΔSSD (having the RGDASYT sequence), rPP-ΔSSD-RGDTSYT, to mimic the porcine PP, and rPP-ΔSSD-RGDDSYT, to mimic the elephant PP, and found that rPP-ΔSSD-RGDDSYT exhibited the most potent cell-adhesive ability followed by rPP-ΔSSD-RGDTSYT. Since the hydropathy index of Ala is 1.8, Thr is −0.7, and Asp is −3.5, the potency of cell adhesion may increase when more hydrophilic amino acid is next to the PP-RGD domain. We also compared rPP-ΔSSD, rPP-ΔSSD-RGDACYT, rPP-ΔSSD-RGDAIYT, and rPP-ΔSSD-RGDAVYT, and found that rPP-ΔSSD-RGDAIYT and rPP-ΔSSD-RGDAVYT exhibited more potent cell-adhesive abilities. This result indicated that the existence of a non-polar hydrophobic amino acid next to Ala^482^ appeared to be more effective for activating the PP-RGD domain. rPP-ΔSSD-RGDNPYT, which had Asn and Pro next to the RGD domain to mimic the human and mouse DMP1-RGD domains, was also able to aid cell adhesion; however, MG63 cells were unable to attach to wells coated with lower than 250 nM of rPP-ΔSSD-GDNPYT. Since rC-DMP-1 exhibited cell-adhesive potency when wells were coated at concentrations of 20 and 100 nM (Fig. 3), the cell-adhesive potency of rPP-ΔSSD-GDNPYT was not as strong as that of rC-DMP-1. These results indicated that other flanking amino acids also influenced sequestration of the cell-adhesive ability of the PP-RGD domain. However, most importantly, a mutation in the 1^st^ and/or 2^nd^ amino acids next to the PP-RGD domain was sufficient to confer cell-adhesive potency to PP.

An evolutionary analysis of the DSPP genomic sequence indicated that ancestor DSPP was created by a DMP-1 duplication [23]. In contrast to C-DMP-1, neither primate nor mouse PP was able to act on the integrin receptor through their RGD domains; therefore, we assumed that PP lost its RGD activity during the evolutionary process after the generation of ancestor DSPP. The PP-RGD domain is not ubiquitously conserved between toothed animals (e. g. rat PP lacks the RGD domain.). Thus, the PP-RGD domain may not play a significant role in tooth development.

A recent *in vitro* study suggested that the inhibition of endogenous *DSPP* expression repressed the carcinogenesis features of human oral squamous cells [46], and the expression of *DSPP* was recently shown to be upregulated in several cancer tissues [4], [47], [48]. Aberrant conditions such as the release of intracellular proteases from cancer cells or a local low pH environment may degrade PP to generate the PP fragments included the RGD domain with an open amino- or carboxyl-terminal side. The integrin accessibility of one of the SIBLINGs, osteopontin, was shown to be enhanced by multiple juxtapositions in RGD amino acid cleavage by plasmin and cathepsin D [49]. A search of the MEROPS (http://merops.sanger.ac.uk/index.shtml), an information resource for peptidases, revealed that more than 80 kinds of peptidase, including cathepsins such as cathepsin D, B, and S, cleaved their substrates at the Ala-Ser bond. Therefore, we incubated 5 µg of rPP with purified cathepsin D, B, and S (sigma) (substrate: enzyme  = 75∶1) for 3 hrs at 37°C in 10 mM sodium acetate buffer at an appropriate pH (pH 3.3 for cathepsin D, 6.0 for cathepsin B, and 6.5 for cathepsin S), and determined whether rPP was cleaved by these enzymes; however, no visible cleaved products were observed by Stains-all staining (data not shown). The functional importance of PP-RGD domains has not yet been evaluated *in vivo*. DMP-1 is known to contribute to the invasion of colon cancer cells in a RGD-independent manner by bridging MMP-9 to CD44 [50]. Therefore, PP may possess such RGD-independent effects as features for carcinogenesis. Future studies to explore degraded PP fragments and the proteases responsible are warranted in order to determine the post-cleavage functions of PP in carcinogenesis as well as hard tissue development.

## Materials and Methods

### Ethics statement

Animal experiments to collect protein samples from DSPP-null and wild type mice were performed in compliance with the Hiroshima University guidelines on the care and use of laboratory animals. All experimental procedures were approved by the Committee of Research Facilities for Laboratory Animal Science of Hiroshima University (Permit Number: A10-81-2). The generation of three human dental pulp cell lines was performed in compliance with the Hiroshima University ethical guidelines for epidemiological research. All experimental procedures were approved by the Committee of Research Ethics of Hiroshima University (Permit Number: D88-3).

### Reagents

Human vitronectin was obtained from BD (Bedford, MA). The neutralizing antibodies against integrin αvβ3 (LM609) and αvβ5 (P1F6) were obtained from Millipore Corporation (Bedford, MA) and the antibody against integrin β1 (4B7) was obtained from Calbiochem (San Diego, CA). Biotinylated-GRGDS (GRGDS, LC-biotin labeled) and Cyclo-(GRGDSP) were purchased from AnaSpec (San Jose, CA). Fifteen kinds of customized peptides were synthesized and>95% purity was examined by Operon Biotechnologies Japan (Tokyo, Japan) or PH Japan (Hiroshima, Japan). Recombinant integrin αvβ3 and αvβ5 were purchased from R&D Systems (Minneapolis MN). The rabbit polyclonal antibody against integrin αv (C2C3) was purchased from GeneTex (Irvine, CA).

### Anti-PP antibody preparation

An affinity-purified rabbit anti-PP polyclonal antibody was generated using a custom antibody preparation service (Operon Biotechnologies Japan). The carboxyl-terminal amino acid sequences to be used as antigens were selected based on low sequence similarities. Japanese white rabbits were primed by an intradermal injection of the antigen peptide (0.2 mg) 3 times at intervals of one week. After 3 weeks of booster injections, 0.025 mg of the same antigen was primed by an intravenous injection to rabbits 3 times at intervals of one week. These rabbits were killed 1 week later, and antisera were harvested from the carotid artery. Antisera were then loaded onto an antigen-binding column and the rabbit anti-PP antibody was eluted. This eluent was used as an affinity-purified rabbit anti-PP antibody. The column flow-through solution was also collected for ELISA.

### ELISA

Ninety-six-well, flat-bottomed microtiter plates (Immulon 4HBX, Thermo Fisher Scientific, Waltham, MA) were coated with either 5 µg/ml antigen peptide in DPBS or DPBS only overnight at 4°C. Plates were washed 3 times with 0.1% Tween 20-DPBS (T-DPBS) and then blocked with 1% BSA in DPBS for 2 hr at room temperature. Plates were washed 5 times with T-DPBS and incubated with the rabbit anti-PP antibody, whole antisera (without antigen-affinity purification), or flow-through solution in triplicate in a serial dilution for 90 min at room temperature. After washing, the wells were incubated with goat anti-rabbit IgG antibodies conjugated with HRP in DPBS (1∶3000) for 1 hr at room temperature. They were washed and detected with 3, 3, 5, 5′-Tetramethyl Benzidine substrate solution (TMB, Pierce, Rockford, IL) for 30 min at room temperature. After the addition of 2 N HCl to stop the colorimetric reaction, optical density was measured at 450 nm using a microtiter plate reader (Multiskan, Thermo Scientific Japan, Tokyo, Japan).

### Dot-blot for the PP antibody

Dentin extracts were collected from wild type and DSPP-null mice as described previously (Suzuki et al., 2009). Mice were euthanized by CO_2_ inhalation, and the molars were extracted. Extracted molars were crushed using a mortar and pestle, and incubated with 4 mol guanidine HCl in the presence of Complete mini EDTA-free protease inhibitor (Roche, Alameda, CA) for 24 hr at RT in order to remove proteins not incorporated into the mineralized matrix. The residue was then demineralized with 4 mol guanidine HCl and 0.5 mol EDTA plus protease inhibitors for 2 days at RT. The solution containing proteins incorporated into the mineralized matrix was dialyzed against 4 mol guanidine HCl for 1 day, then against distilled water for another 2 days. The dialyzed solution was lyophilized and dissolved into distilled water containing a protease inhibitor. Five micrograms in 20 µl of the dentin extracts from wild type and DSPP-null mice and BSA were dot-blotted onto a nitrocellulose membrane with a dot-blot apparatus. The membrane was blocked with a solution of 3% non-fat dried milk in DPBS solution, followed by incubation with the rabbit anti-PP antibody (1∶2500) for 1 hr at room temperature. After washing the membrane with DPBS buffer, it was incubated with the goat anti-rabbit antibody conjugated with HRP in DPBS (1∶10,000) for 1 hr at room temperature, and developed using the chemiluminescent substrate (WesternBright ECL, Advansta, Menlo Park, CA).

### SDS-PAGE, gel staining, and western blotting

All protein samples were reduced with DTT and loaded onto NuPAGE Bis-Tris (Life Technologies) in MOPS buffer. Separated proteins were transferred onto a PVDF membrane for immunodetection. The membrane was blocked with a PVDF blocking agent (TOYOBO, Tokyo, Japan) for 1 hr at room temperature, and was then incubated with the anti-PP antibody (1∶2500) for 1 hr at room temperature. After washing the membrane with T-DPBS, it was incubated with the HRP-conjugated goat anti-rabbit antibody (1∶20,000) as the secondary antibody for 1 hr at room temperature. Immunoreactive proteins were identified using a chemiluminescent substrate (WesternBright ECL). To efficiently detect acidic proteins, the gel was washed and fixed with 25% isopropanol for 3 hr. The fixative solution was replaced every 20 min. The gel was stained overnight with 0.025% Stains-All (Sigma) containing 30 mM Tris-HCl, 7.5% formamide, and 25% isopropanol at pH 8.8 and then washed with deionized water until the protein bands were visible. Coomassie brilliant blue staining was performed with Coomassie brilliant blue R (Sigma) following a general protocol.

### Preparation and purification of recombinant proteins

Mouse PP (from the Asp^452^ to the stop codon) was amplified from mouse DSPP cDNA obtained from mouse incisors and cloned into a pBS-sk(+) vector (Stratagene, La Jolla, CA). Since the long-repeat nucleotide sequence-coding SSD repeats in PP were easily released from circular DNA during the transformation process, PP nucleotide sequences were cloned following a previously described procedure [6]. PP nucleotide sequences was cloned into the pCEP4-Mul-PURD expression vector (a kind gift from Dr. Yoshi Yamada, NIDCR/NIH) [51] such that the amino-terminus of recombinant proteins was 6× His-tagged.

The PP vector was transfected into 293EBNA cells utilizing X-tremeGENE (Roche, Indianapolis, IN) and incubated for 24 hr. The transfection medium was replaced with growth medium containing puromycin (5 µg/ml), and transfected cells were then cultured for 3 days. Surviving cells were routinely cultured with puromycin (0.5 µg/ml) and used in the expression of the His-tagged recombinant PP (rPP) protein. To collect supernatants containing recombinant proteins, these puromycin-resistant cells were cultured with growth medium containing 10% fetal bovine serum (FBS) until confluent. The medium was replaced with new growth medium containing 5% FBS and 1× Insulin-Transferrin-Selenium (ITS) (BD Biosciences) and then cultured for 3 days. The growth medium was replaced with serum-free collection medium containing 1× ITS and then cultured for 5 days. The collection medium was collected and replaced every 24 hr during a 5-day period. Recombinant proteins were purified from the collection medium as follows. All procedures were generally performed in a cold room (4°C). Non-serum medium containing recombinant proteins was added to anion-exchange columns (Q Sepharose Fast Flow, GE Healthcare Japan, Tokyo, Japan) equilibrated with 20 mM Tris-HCl (pH 7.0), and these columns were then washed completely with 0.3 M NaCl/20 mM Tris-HCl buffer. Recombinant proteins were eluted from these columns with 0.6 M NaCl/20 mM Tris-HCl buffer. The eluents were further mixed with an equal amount of 20 mM Tris-HCl to decrease the NaCl concentration to 0.3 M. Solutions containing recombinant proteins in 0.3 M NaCl/20 mM Tris-HCl buffer were then added to the next anion-exchange columns (Hi Trap Q column, GE Healthcare Japan), which had been washed completely with 0.3 M NaCl/20 mM Tris-HCl buffer. Recombinant proteins were eluted from the columns with 0.36 M NaCl/20 mM Tris-HCl buffer. A NaCl concentration of 0.36 M was preliminarily decided to be suitable for eluting the maximum amount of recombinant proteins with the minimum amount of contamination by other proteins. Eluents were then dialyzed against His-binding buffer (50 mM NaH_2_PO_4_, 150 mM NaCl, 10 mM imidazole, pH 8.0), incubated with TALON Magnetic Beads (Clontech Inc, Mountain View, CA) to ultimately purify the proteins via the chelation of His-tags and cobalt ions, and finally eluted with 250 mM imidazole/DPBS. These eluents were dialyzed against DPBS and lyophilized.

Using repeat sequence exclusion during transformation into *E. coli*, we transformed the PP pBS-sk (+) vector into HST08-competent cells (Takara Bio Inc., Otsu, Japan) with a general protocol and obtained the PP-ΔSSD vector, in which SSD repeats were flipped. Using the PP pBS-sk(+) vector, nucleotide sequences encoding Asp^481^ were replaced with nucleotides encoding Glu to generate the PP-RGE pBS-sk(+) vector with a site-directed mutagenesis kit (Stratagene). Using the PP-ΔSSD pBS-sk(+) vector, nucleotide sequences encoding Ala^482^ were replaced with nucleotides encoding Thr and Asp to generate the PP-ΔSSD-RGDTSYT and PP-ΔSSD-RGDDSYT pBS-sk(+) vectors, respectively, nucleotide sequences encoding Ser^483^ were replaced with nucleotides encoding Cys, Ile, and Val to generate the PP-ΔSSD-RGDACYT, PP-ΔSSD-RGDAIYT, and PP-ΔSSD-RGDAVYT pBS-sk(+) vectors, respectively, and nucleotide sequences encoding Ala^482^ and Ser^483^ were replaced with nucleotides encoding Asn and Pro to generate the PP-ΔSSD-RGDNPYT pBS-sk(+) vector with a site-directed mutagenesis kit. Using the PP and PP-RGE pBS-sk(+) vectors, nucleotide sequences encoding Asp^452^ to Ala^482^ were amplified to generate the PP-(Ala terminal) and PP-RGE-(Ala terminal) pBS-sk(+) vectors, respectively. The mouse C-DMP-1 (from Asp^213^ to the stop codon: the full-length of the carboxyl-terminal of DMP-1) sequence was amplified from MC3T3-E1 cell cDNA and cloned into the pBS-sk(+) vector. The recombinant PP-ΔSSD (rPP-ΔSSD), PP-ΔSSD-RGDTSYT (rPP-ΔSSD-RGDTSYT), PP-ΔSSD-RGDDSYT (rPP-ΔSSD-RGDDSYT), PP-ΔSSD-RGDACYT (rPP-ΔSSD-RGDACYT), PP-ΔSSD-RGDAIYT (rPP-ΔSSD-RGDAIYT), PP-ΔSSD-RGDAVYT (rPP-ΔSSD-RGDAVYT), PP-ΔSSD-RGDNPYT (rPP-ΔSSD-RGDNPYT), PP-RGE (rPP-RGE), PP-(Ala terminal) (rPP-(Ala terminal)), PP-RGE-(Ala terminal) (rPP-RGE-(Ala terminal)), and C-DMP-1 (rC-DMP-1) proteins were generated in a similar manner to rPP, as described above. Since rPP-ΔSSD, rPP-ΔSSD-RGDTSYT, rPP-ΔSSD-RGDDSYT, rPP-ΔSSD-RGDACYT, rPP-ΔSSD-RGDAIYT, rPP-ΔSSD-RGDAVYT, rPP-ΔSSD-RGDNPYT, rPP-(Ala terminal), rPP-RGE-(Ala terminal), and rC-DMP-1 were efficiently secreted into the supernatant of transfected 293EBNA cells, the eluents from columns with 0.6 M NaCl/20 mM Tris-HCl buffer were directly dialyzed against His-binding buffer and then purified with TALON magnetic beads. The protein concentrations of the recombinant proteins were determined using a BCA kit (Pierce).

### Cell culture

The mouse pre-osteoblastic cell line MC3T3-E1 (subclone 4) and mouse myoblast cell line C2C12 were purchased from the ATCC (Rockville, MD). The human osteosarcoma cell lines MG63 and Saos2, and parental heterogeneous MC3T3-E1 were purchased from the RIKEN Cell Bank (Tsukuba, Japan). Human dental pulp (hDPC) cells from three different patients were isolated from healthy teeth extracted for orthodontic purposes with informed consent. The pulp chamber was opened by minimum drilling and pulp tissue was removed from the chamber using a barbed broach. The extracted tissue was placed onto a culture dish plate and cells that were out-growths from the tissue were used as dental pulp cells. MC3T3-E1 cells, both subclone 4 and heterogeneous cells, were maintained in alpha MEM (Life Technologies). 293EBNA, MG63, 3 different kinds of hDPC, and C2C12 cells were maintained in DMEM (Life Technologies). Saos2 cells were maintained in McCoy's medium (Life Technologies). All media were supplemented with 100 units/ml penicillin, 100 µg/ml streptomycin (Life Technologies), and 10% FBS, except for McCoy's medium, which was supplemented with 15% FBS. All cells were cultivated at 37°C under humidified 5% CO_2_, and 95% air atmospheric conditions.

### Cell adhesion assay

Fifty microliters of a 20 or 100 nM solution of recombinant proteins or vitronectin was coated onto a 96-well plate (Immulon 4HBX) in DPBS overnight at 4°C. The wells were then rinsed twice with DPBS and blocked with 1% BSA in DPBS for 1 hr at 37°C, followed by 3 washes with DPBS. Subconfluent cells were rinsed twice with DPBS, harvested using 5 mM EDTA in DPBS for 20 min at room temperature, collected by centrifugation, and then suspended in serum-free RPMI 1640 (Life Technologies) with or without divalent cations as noted. Suspended cells were preincubated with 1 mM of the various peptides for 10 min at 37°C for blocking experiments. These cells were seeded onto wells at a density of 5×10^4^ cells/well in 50 µl of serum-free medium. After a 1-hr incubation at 37°C, the wells were rinsed 3 times with serum-free medium to remove non-adherent cells. Adherent cells were fixed with 3.7% formaldehyde for 10 min and then stained with 0.2% crystal violet in 10% ethanol for 10 min at room temperature. Wells were rinsed several times with water and dried overnight. The dye was then solubilized with 150 µl of 1% SDS and quantified by measuring absorbance at 570 nm on a microtiter plate reader (Multiskan).

### Solid phase binding assay

Ninety-six-well plates (Immulon 4HBX) were coated with rPP, rPP-ΔSSD, rPP-RGE, rC-DMP-1, and vitronectin and then blocked as described in the Cell adhesion assay section, followed by rinsing twice with binding buffer (50 mM Tris, 150 mM NaCl, 1 mM MnCl2). Various amounts (0∼400 ng) of recombinant integrin αvβ3 and αvβ5 in 50 µl of binding buffer were then added to the coated wells and incubated overnight at 4°C. To immunologically detect bound αvβ3 and αvβ5, the wells were rinsed twice with 0.1% Tween-binding buffer (T-binding buffer) and incubated with the rabbit anti integrin αv antibody (C2C3) (1∶1000) for 90 min at room temperature. After washing twice with T-binding buffer, the wells were incubated with goat anti-rabbit IgG antibodies conjugated with HRP in binding buffer (1∶1000) for 1 hr at room temperature. The wells were then rinsed twice with 0.1% Tween-binding buffer and twice with binding buffer, and detected with TMB for 30 min at room temperature. After the addition of 2N HCl to stop the colorimetric reaction, optical density was measured at 450 nm using a microtiter plate reader (Multiskan).

### Integrin competitive binding assay

The wells of 96-well plates (Immulon 4HBX) were coated with 0.05 µg/well recombinant integrin αvβ3 overnight at 4°C. The coated plates were washed twice with T-binding buffer and blocked with 1% BSA in binding buffer for 1 hr at 37°C. The wells were then washed twice with T-binding buffer and incubated with 5 nM biotinylated-GRGDS peptide with or without various peptides or cyclic-(GRGDSP) for 3 hr at room temperature. The wells were rinsed twice with T-binding buffer, and then incubated with the streptavidin-HRP polymer (Sigma-Aldrich) (1∶5,000) for 1 hr at room temperature to detect bound biotinylated-GRGDS. The wells were rinsed twice with T-binding buffer and twice with binding buffer, and detected with TMB for 30 min at room temperature. After the addition of 2N HCl to stop the colorimetric reaction, optical density was measured at 450 nm using a microtiter plate reader (Multiskan).

### rPP-ΔSSD and rPP-(Ala terminal) binding assay

The Alexa Fluor 488 tetrafluorophenyl ester (Life Technologies) was mixed with PP-ΔSSD and rPP-(Ala terminal) at a dye: protein molar ratio  = 30: 1 for 1 hr at room temperature in DPBS containing 0.1 M sodium bicarbonate. The conjugated samples were extensively dialyzed against DPBS to remove unconjugated dye. The protein concentration of Alexa Fluor 488 conjugated-PP-ΔSSD (Alexa-PP-ΔSSD) and Alexa Fluor 488 conjugated-rPP-(Ala terminal) (Alexa-rPP-(Ala terminal)) was determined using a BCA kit. One hundred microliters of 15.625, 62.5, and 250 nM of Alexa-PP-ΔSSD and Alexa-rPP-(Ala terminal) were added onto black 96-well plates and fluorescence intensity was measured by ARVO X One (PerkinElmer Japan, Kanagawa, Japan) to analyze the degree of labeling. In the cytofluorometric assessment, MG63 cells were harvested as described for the cell adhesion assay and then suspended in 0.5% BSA in DMEM with 2 mM MnCl_2._ Suspended cells were incubated with soluble Alexa-rPP-ΔSSD and Alexa-rPP-(Ala terminal) (250 nM) for 1 hr at 4°C. Cell pellets were washed twice with DPBS containing 2% FBS with 200 nM MnCl_2_ and the binding of soluble Alexa Fluor 488-rPP-ΔSSD and rPP-(Ala terminal) was then determined by flow cytometry.

### Immunofluorescence analysis for the actin cytoskeleton

Thirty-five-millimeter glass dishes (Matsunami, Japan) were coated with 100 nM solution of rPP, rPP-(Ala terminal), rPP-RGE-(Ala terminal), or rC-DMP1 in DPBS overnight at 4°C. The wells were then rinsed twice with DPBS and blocked with 1% BSA in DPBS for 1 hr at 37°C, followed by 3 washes with DPBS. Cells were harvested as described for the cell adhesion assay and then suspended in serum-free RPMI 1640 with 1 mM MnCl_2_. Cells were seeded in serum-free medium and incubated at 37°C for 1 hr. Cells were then washed twice with DPBS, fixed with 2.5% paraformaldehyde, permeabilized with 0.5% Triton X-100, and then incubated with Actin-stain 488 phalloidin (Cytoskeleton, Inc., Denver, CO) for 30 min. Actin staining was observed using a Leica microscope and images were captured with a digital camera.

### Cell migration assay

Haptotaxis cell migration was assayed with Transwell migration chambers, the pore size of which was 8 µm (353097 cell culture-treated, Falcon System Inc., Columbia, MD). The undersides of the membranes were coated at room temperature overnight with 100 µl of a 1 µM solution of recombinant proteins followed by 2 washes with DPBS. MG63 cells were harvested as described for the cell adhesion assay and then suspended in 0.1% BSA in RPMI 1640. These cells were seeded onto the upper chamber at a density of 1×10^5^ cells/well in 150 µl of 0.1% BSA in RPMI 1640. The lower chamber was filled with 600 µl of 0.1% BSA in RPMI 1640. After a 12-hr incubation at 37°C, the membranes were rinsed twice with warmed DPBS, fixed with 3.7% formaldehyde for 10 min, and then stained with 0.2% crystal violet in 10% ethanol for 10 min at room temperature. Non-migrated cells were then removed from the upper surface of the membranes with a damp cotton swab. Cells that had migrated were counted using 10 randomly selected fields at 200× magnification.

### Statistical Analysis

Statistical analysis was performed by a one-way analysis of variance (ANOVA), followed by Dunnett's test for Figures 3, 4, 5, 6, 7D, 8, and 9 and by Tukey's test for Figures 7A, B, and C. Standard samples of the posthoc test have been described in each of the figure legends.

## References

[1] Butler WT, Ritchie H (1995) The nature and functional significance of dentin extracellular matrix proteins. Int J Dev Biol 39: 169–179. 7626404

[2] Fisher LW, Torchia DA, Fohr B, Young MF, Fedarko NS (2001) Flexible Structures of SIBLING Proteins, Bone Sialoprotein, and Osteopontin. Biochem Biophys Res Commun 280: 460–465. 11162539 10.1006/bbrc.2000.4146

[3] Fisher LW, Fedarko NS (2003) Six genes expressed in bones and teeth encode the current members of the SIBLING family of proteins. Connect Tissue Res 44: 33–40. 12952171

[4] Bellahcene A, Castronovo V, Ogbureke KU, Fisher LW, Fedarko NS (2008) Small integrin-binding ligand N-linked glycoproteins (SIBLINGs): multifunctional proteins in cancer. Nat Rev Cancer 8: 212–226. 18292776 10.1038/nrc2345 PMC2484121

[5] Jonsson M, Fredriksson S (1978) Isoelectric focusing of the phosphoprotein of rat incisor dentin in ampholine and acid pH gradients. Evidence for carrier ampholyte protein complexes. J Chromatogr 21: 234–242. 10.1016/s0021-9673(00)92338-0 29908

[6] McKnight DA, Suzanne HP, Hart TC, Hartsfield JK, Wilson A, et al (2008) A comprehensive analysis of normal variation and disease-causing mutations in the human DSPP gene. Hum Mutat 29: 1392–1404. 18521831 10.1002/humu.20783 PMC5534847

[7] Qin C, Brunn JC, Cadena E, Ridall A, Tsujigiwa H, et al (2002) The expression of dentin sialophosphoprotein gene in bone. J Dent Res 81: 392–394. 12097430 10.1177/154405910208100607

[8] Qin C, Brunn JC, Cadena E, Ridall A, Butler WT (2003) Dentin sialoprotein in bone and dentin sialophosphoprotein gene expressed by osteoblasts. Connect Tissue Res 44: 179–183. 12952194

[9] Ogbureke KU, Fisher LW (2004) Expression of SIBLINGs and their partner MMPs in salivary glands. J Dent Res 83: 664–670. 15329369 10.1177/154405910408300902

[10] Alvares K, Kanwar YS, Veis A (2006) Expression and potential role of dentin phosphophoryn (DPP) in mouse embryonic tissues involved in epithelial-mesenchymal interactions and branching morphogenesis. Dev Dyn 235: 2980–2990. 16937369 10.1002/dvdy.20935

[11] Alvares K, Stern PH, Veis A (2013) Dentin phosphoprotein binds annexin 2 and is involved in calcium transport in rat kidney ureteric bud cells. J Biol Chem 288: 13036–13045. 23525114 10.1074/jbc.M112.389627 PMC3642346

[12] Sreenath T, Thyagarajan T, Hall B, Longenecker G, D'Souza R, et al (2003) Dentin sialophosphoprotein knockout mouse teeth display widened predentin zone and develop defective dentin mineralization similar to human dentinogenesis imperfecta type III. J Biol Chem 278: 24874–24880. 12721295 10.1074/jbc.M303908200

[13] Verdelis K, Ling Y, Sreenath T, Haruyama N, Macdougall M, et al (2008) DSPP effects on in vivo bone mineralization. Bone 43: 983–990. 18789408 10.1016/j.bone.2008.08.110 PMC2621360

[14] Suzuki S, Sreenath T, Haruyama N, Honeycutt C, Terse A, et al (2009) Dentin sialoprotein and dentin phosphoprotein have distinct roles in dentin mineralization. Matrix Biol 28: 221–235. 19348940 10.1016/j.matbio.2009.03.006 PMC2758621

[15] Sfeir C, Lee D, Li J, Zhang X, Boskey AL, et al (2011) Expression of phosphophoryn is sufficient for the induction of matrix mineralization by mammalian cells. J Biol Chem 286: 20228–20238. 21343307 10.1074/jbc.M110.209528 PMC3121506

[16] Ye L, MacDougall M, Zhang S, Xie Y, Zhang J, et al (2004) Deletion of dentin matrix protein-1 leads to a partial failure of maturation of predentin into dentin, hypomineralization, and expanded cavities of pulp and root canal during postnatal tooth development. J Biol Chem 279: 19141–19148. 14966118 10.1074/jbc.M400490200

[17] Yamakoshi Y, Hu JC, Fukae M, Iwata T, Kim JW, et al (2005) Porcine dentin sialoprotein is a proteoglycan with glycosaminoglycan chains containing chondroitin 6-sulfate. J Biol Chem 280: 1552–1560. 15537641 10.1074/jbc.M409606200

[18] Qin C, Huang B, Wygant JN, McIntyre BW, McDonald CH, et al (2006) A chondroitin sulfate chain attached to the bone dentin matrix protein 1 NH2-terminal fragment. J Biol Chem 281: 8034–8040. 16421105 10.1074/jbc.M512964200

[19] Tsuchiya S, Simmer JP, Hu JC, Richardson AS, Yamakoshi F, et al (2010) Astacin proteases cleave dentin sialophosphoprotein (Dspp) to generate dentin phosphoprotein (Dpp). J Bone Miner Res 26: 220–228. 10.1002/jbmr.202 PMC3179315 20687161

[20] Deshpande AS, Fang PA, Zhang X, Jayaraman T, Sfeir C, et al (2011) Primary Structure and phosphorylation of Dentin Matrix Protein 1 (DMP1) and Dentin Phosphophoryn (DPP) uniquely determine their role in biomineralization. Biomacromolecules 12: 2933–2945. 21736373 10.1021/bm2005214 PMC3171794

[21] Suzuki S, Haruyama N, Nishimura F, Kulkarni AB (2012) Dentin sialophosphoprotein and dentin matrix protein-1: Two highly phosphorylated proteins in mineralized tissues. Arch Oral Biol 57: 1165–1175. 22534175 10.1016/j.archoralbio.2012.03.005 PMC3517212

[22] Gibson MP, Zhu Q, Wang S, Liu Q, Liu Y, et al (2013) The rescue of dentin matrix protein 1 (DMP1)-deficient tooth defects by the transgenic expression of dentin sialophosphoprotein (DSPP) indicates that DSPP is a downstream effector molecule of DMP1 in dentinogenesis. J Biol Chem 288: 7204–7214. 23349460 10.1074/jbc.M112.445775 PMC3591629

[23] McKnight DA, Fisher LW (2009) Molecular evolution of dentin phosphoprotein among toothed and toothless animals. BMC Evol Biol 9: 299. 20030824 10.1186/1471-2148-9-299 PMC2803795

[24] von Marschall Z, Fisher LW (2008) Dentin matrix protein-1 isoforms promote differential cell attachment and migration. J Biol Chem 283: 32730–32740. 18819913 10.1074/jbc.M804283200 PMC2583300

[25] von Marschall Z, Fisher LW (2010) Dentin sialophosphoprotein (DSPP) is cleaved into its two natural dentin matrix products by three isoforms of bone morphogenetic protein-1 (BMP1). Matrix Biol 29: 295–303. 20079836 10.1016/j.matbio.2010.01.002 PMC2862847

[26] Sun Y, Lu Y, Chen S, Prasad M, Wang X, et al (2010) Key proteolytic cleavage site and full-length form of DSPP. J Dent Res 89: 498–503. 20332332 10.1177/0022034510363109 PMC2873034

[27] Ritchie HH, Yee CT, Tang XN, Dong Z, Fuller RS (2012) DSP-PP precursor protein cleavage by tolloid-related-1 protein and by bone morphogenetic protein-1. PLoS One 7: e41110. 22815932 10.1371/journal.pone.0041110 PMC3398931

[28] Yang RT, Lim GL, Dong Z, Lee AM, Yee CT, et al (2013) The efficiency of dentin sialoprotein-phosphophoryn processing is affected by mutations both flanking and distant from the cleavage site. J Biol Chem 288: 6024–6033. 23297400 10.1074/jbc.M112.382952 PMC3581424

[29] Yamakoshi Y, Lu Y, Hu JC, Kim JW, Iwata T, et al (2008) Porcine dentin sialophosphoprotein: length polymorphisms, glycosylation, phosphorylation, and stability. J Biol Chem 283: 14835–14844. 18359767 10.1074/jbc.M800633200 PMC3762552

[30] Yamakoshi Y (2009) Dentinogenesis and Dentin Sialophosphoprotein (DSPP). J Oral Biosci 51: 134. 20157636 10.2330/joralbiosci.51.134 PMC2821091

[31] Hansen JC, Lu X, Ross ED, Woody RW (2006) Intrinsic protein disorder, amino acid composition, and histone terminal domains. J Biol Chem 281: 1853–1856. 16301309 10.1074/jbc.R500022200

[32] Suskiewicz MJ, Sussman JL, Silman I, Shaul Y (2011) Context-dependent resistance to proteolysis of intrinsically disordered proteins. Protein Sci. 20: 1285–1297. 10.1002/pro.657 PMC3189518 21574196

[33] Hayman EG, Pierschbacher MD, Ohgren Y, Ruoslahti E (1983) Serum spreading factor (vitronectin) is present at the cell surface and in tissues. Proc Natl Acad Sci U S A 80: 4003–4007. 6191326 10.1073/pnas.80.13.4003 PMC394188

[34] Ruoslahti E, Pierschbacher MD (1987) New perspectives in cell adhesion: RGD and integrins. Science 238: 491–497. 2821619 10.1126/science.2821619

[35] Wayner EA, Orlando RA, Cheresh DA (1991) Integrins alpha v beta 3 and alpha v beta 5 contribute to cell attachment to vitronectin but differentially distribute on the cell surface. J Cell Biol 113: 919–929. 1709170 10.1083/jcb.113.4.919 PMC2288998

[36] Ruoslahti E (1996) RGD and other recognition sequences for integrins. Annu Rev Cell Dev Biol 12: 697–715. 8970741 10.1146/annurev.cellbio.12.1.697

[37] Felding-Habermann B, O'Toole TE, Smith JW, Fransvea E, Ruggeri ZM, et al (2001) Integrin activation controls metastasis in human breast cancer. Proc Natl Acad Sci USA 98: 1853–1858. 11172040 10.1073/pnas.98.4.1853 PMC29346

[38] Xiong JP, Stehle T, Diefenbach B, Zhang R, Dunker R, et al (2001) Crystal structure of the extracellular segment of integrin alpha V beta3. Science 294: 339–345. 11546839 10.1126/science.1064535 PMC2885948

[39] Xiong JP, Stehle T, Zhang R, Joachimiak A, Frech M, et al (2002) Crystal structure of the extracellular segment of integrin alpha V beta3 in complex with an Arg-Gly-Asp ligand. Science 296: 151–155. 11884718 10.1126/science.1069040

[40] Mould AP, Akiyama SK, Humphries MJ (1995) Regulation of integrin alpha 5 beta 1-fibronectin interactions by divalent cations. Evidence for distinct classes of binding sites for Mn2+, Mg2+, and Ca2+. J Biol Chem 270: 26270–26277. 7592835 10.1074/jbc.270.44.26270

[41] Stuiver I, Ruggeri Z, Smith JW (1996) Divalent cations regulate the organization of integrins alpha v beta 3 and alpha v beta 5 on the cell surface. J Cell Physiol 168: 521–531. 8816906 10.1002/(SICI)1097-4652(199609)168:3<521::AID-JCP4>3.0.CO;2-R

[42] Eapen A, Ramachandran A, George A (2012) Dentin phosphoprotein (DPP) activates integrin-mediated anchorage-dependent signals in undifferentiated mesenchymal cells. J Biol Chem 287: 5211–5224. 22134916 10.1074/jbc.M111.290080 PMC3285302

[43] Eapen A, Kulkarni R, Ravindran S, Ramachandran A, Sundivakkam P, et al (2013) Dentin phosphophoryn activates Smad protein signaling through Ca2+-calmodulin-dependent protein kinase II in undifferentiated mesenchymal cells. J Biol Chem 288: 8585–8595. 23362283 10.1074/jbc.M112.413997 PMC3605677

[44] Ravindran S, Snee PT, Ramachandran A, George A (2013) Acidic domain in dentin phosphophoryn facilitates cellular uptake: implications in targeted protein delivery. J Biol Chem 288: 16098–16109. 23589294 10.1074/jbc.M113.450585 PMC3668765

[45] Yasuda Y, Izumikawa M, Okamoto K, Tsukuba T, Saito T (2008) Dentin phosphophoryn promotes cellular migration of human dental pulp cells. J Endod 34: 575–578. 18436037 10.1016/j.joen.2008.02.018

[46] Joshi R, Tawfik A, Edeh N, McCloud V, Looney S, et al (2010) Dentin sialophosphoprotein (DSPP) gene-silencing inhibits key tumorigenic activities in human oral cancer cell line, OSC2. PLoS One 5: e13974. 21103065 10.1371/journal.pone.0013974 PMC2980487

[47] Fisher LW, Jain A, Tayback M, Fedarko NS (2004) Small integrin binding ligand N-linked glycoprotein gene family expression in different cancers. Clin Cancer Res 10: 8501–8511. 15623631 10.1158/1078-0432.CCR-04-1072

[48] Jain A, McKnight DA, Fisher LW, Humphreys EB, Mangold LA, et al (2009) Small integrin-binding proteins as serum markers for prostate cancer detection. Clin Cancer Res 15: 5199–5207. 19671866 10.1158/1078-0432.CCR-09-0783 PMC2766346

[49] Christensen B, Schack L, Kläning E, Sørensen ES (2010) Osteopontin is cleaved at multiple sites close to its integrin-binding motifs in milk and is a novel substrate for plasmin and cathepsin D. J Biol Chem 285: 7929–7937. 20071328 10.1074/jbc.M109.075010 PMC2832943

[50] Karadag A, Fedarko NS, Fisher LW (2005) Dentin matrix protein 1 enhances invasion potential of colon cancer cells by bridging matrix metalloproteinase-9 to integrins and CD44. Cancer Res 65: 11545–11552. 16357164 10.1158/0008-5472.CAN-05-2861 PMC1350722

[51] Hozumi K, Suzuki N, Nielsen PK, Nomizu M, Yamada Y (2006) Laminin alpha1 chain LG4 module promotes cell attachment through syndecans and cell spreading through integrin alpha2beta1. J Biol Chem 281: 32929–32940. 16945929 10.1074/jbc.M605708200

